# Influences of HLH-2 stability on anchor cell fate specification during *Caenorhabditis elegans* gonadogenesis

**DOI:** 10.1093/g3journal/jkac028

**Published:** 2022-02-03

**Authors:** Justin M Benavidez, Jee Hun Kim, Iva Greenwald

**Affiliations:** Department of Biological Sciences, Columbia University, New York, NY 10027, USA

**Keywords:** HLH-2, gonad, *C. elegans*, ubiquitin ligase, cell fate

## Abstract

The *Caenorhabditis elegans* E protein ortholog HLH-2 is required for the specification and function of the anchor cell, a unique, terminally differentiated somatic gonad cell that organizes uterine and vulval development. Initially, 4 cells—2 α cells and their sisters, the β cells—have the potential to be the sole anchor cell. The β cells rapidly lose anchor cell potential and invariably become ventral uterine precursor cells, while the 2 α cells interact via LIN-12/Notch to resolve which will be the anchor cell and which will become another ventral uterine precursor cell. HLH-2 protein stability is dynamically regulated in cells with anchor cell potential; initially present in all 4 cells, HLH-2 is degraded in presumptive ventral uterine precursor cells while remaining stable in the anchor cell. Here, we demonstrate that stability of HLH-2 protein is regulated by the activity of *lin-12/*Notch in both α and β cells. Our analysis provides evidence that activation of LIN-12 promotes degradation of HLH-2 as part of a negative feedback loop during the anchor cell/ventral uterine precursor cell decision by the α cells, and that absence of *lin-12* activity in β cells increases HLH-2 stability and may account for their propensity to adopt the anchor cell fate in a *lin-12* null background. We also performed an RNA interference screen of 232 ubiquitin-related genes and identified 7 genes that contribute to HLH-2 degradation in ventral uterine precursor cells; however, stabilizing HLH-2 by depleting ubiquitin ligases in a *lin-12(+)* background does not result in supernumerary anchor cells, suggesting that LIN-12 activation does not oppose *hlh-2* activity solely by causing HLH-2 protein degradation. Finally, we provide evidence for *lin-12*-independent transcriptional regulation of *hlh-2* in β cells that correlates with known differences in POP-1/TCF levels and anchor cell potential between α and β cells. Together, our results indicate that *hlh-2* activity is regulated at multiple levels to restrict the anchor cell fate to a single cell.

## Introduction

**Fig. 1.**
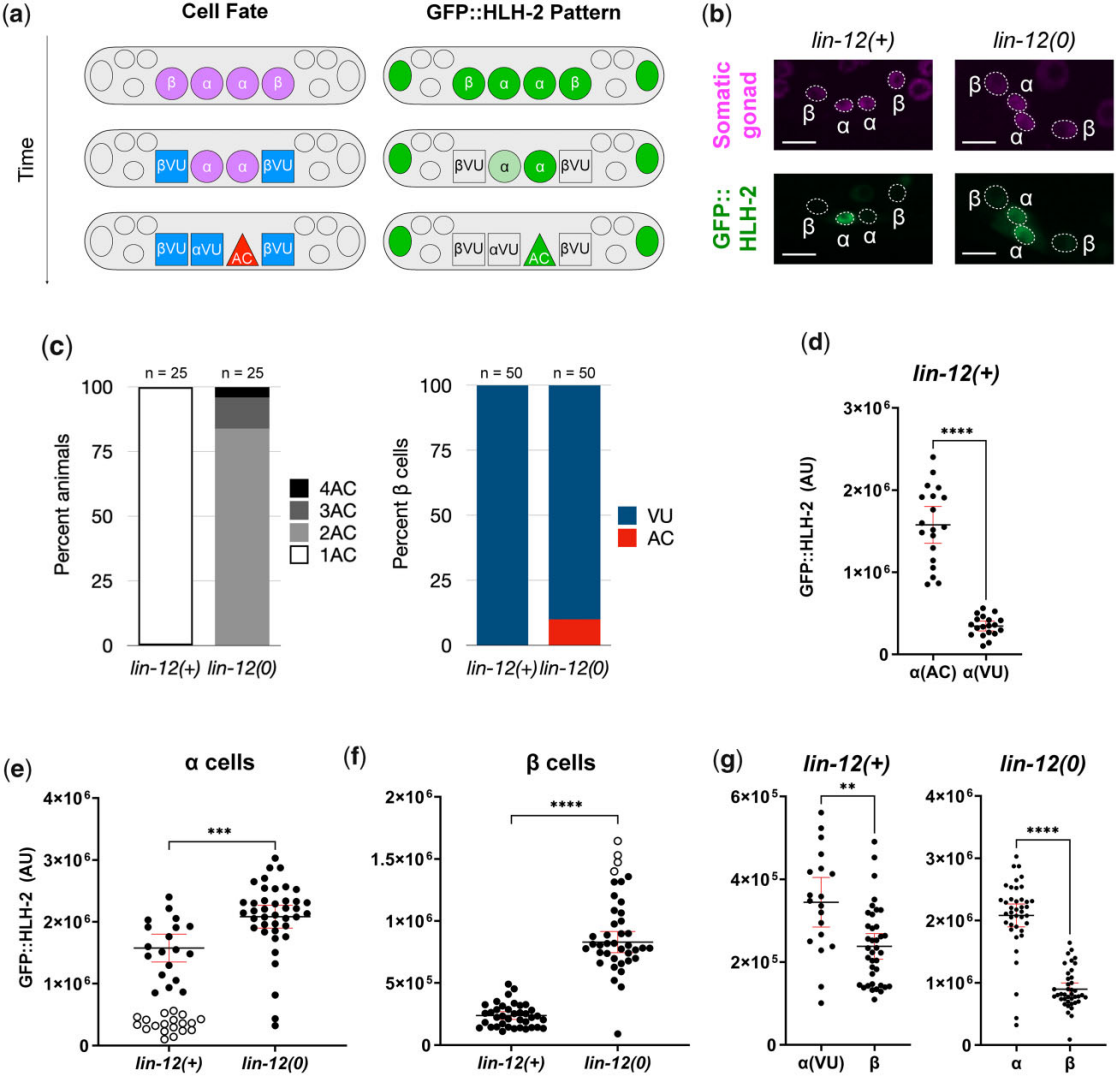
GFP::HLH-2 accumulates to a higher level in α and β cells in a *lin-12(0)* background. a) Schematic representation of the progression of α and β cell specification and the associated pattern of GFP::HLH-2 accumulation. Left, the α and β cells initially have the potential to be an AC; in a *lin-12(+)* background, the β cells rapidly lose this potential, and the α cells maintain it, requiring the *lin-12*-mediated AC/VU decision to resolve which α cell will be the AC. Right, endogenous GFP::HLH-2 is initially visible in the α and β cells, then becomes progressively restricted, first to both α cells, then to the AC. b) Representative photomicrographs showing GFP::HLH-2 levels (green) and expression of a somatic gonad marker, *arTi145[ckb-3p::mCherry-h2b]* (magenta) in *lin-12(+)* and *lin-12(0)* backgrounds. All images are maximum projections of z-stacks collected using a spinning disc confocal microscope. Scale bars are 5 μm. c) Cell fates in *lin-12(+)* and *lin-12(0)* hermaphrodites grown at 20^°^. Left, the proportion of hermaphrodites with the number of ACs indicated; right, the proportion of β cells that adopt AC or VU fates. *n* for each experiment is noted atop each respective bar. As most β cells are VUs, differences in GFP::HLH-2 accumulation between *lin-12(+)* and *lin-12(0)* described in E-G are not due to cell fate transformations, as confirmed by correcting for inferred transformations based on these data as described. d) Comparison of GFP::HLH-2 levels in the inferred presumptive ACs and αVUs of *lin-12(+)*. For this comparison, the cell fates in each individual was inferred based on the differential expression of GFP::HLH-2, with the cell expressing higher levels of GFP in any given individual inferred to be the AC. e) Comparison of GFP::HLH-2 levels in the α cells of *lin-12(+)* and *lin-12(0)*, inferred VUs (white with black outline) were excluded from statistical analysis to avoid comparing cells of different fates. Note that even excluding inferred VUs, *lin-12(0)* ACs still have a higher level of GFP::HLH-2 than *lin-12(+)* ACs. f) Comparison of GFP::HLH-2 levels in the β cells of *lin-12(+)* and *lin-12(0)*. The top 10% of β cells in *lin-12(0)* (white with black outline) are likely to be supernumerary ACs (see C), and were excluded from the statistical analysis. Note that the remaining *lin-12(0)* β cells have a higher level of GFP::HLH-2 than *lin-12(+)* ACs. g) Comparison of α and β cells within *lin-12(+)* (left) or *lin-12(0)* (right) reveals that the difference between α and β cells does not depend on *lin-12* activity. In a *lin-12(0)* background, β cells have a lower level of GFP::HLH-2 than α cells. In a *lin-12(+)* background, αVUs and βVUs both experience *lin-12* activity and resulting degradation of GFP::HLH-2, but α cells still have a higher level of GFP::HLH-2 than β cells. Statistical tests for d–g were Mann–Whitney *U*-tests. **P < 0.01, ***P < 0.001, ****P < 0.0001. Black lines represent the mean GFP::HLH-2 fluorescence, and red error bars are 95% confidence intervals.

The *Caenorhabditis* *elegans* hermaphrodite gonad develops post-embryonically from a gonad primordium that contains 2 somatic progenitors and 2 germline progenitors at hatching (Kimble and Hirsh 1979).  The first phase of *C. elegans* gonadogenesis begins with the division of 2 progenitor cells, Z1 and Z4, during the first larval (L1) stage, and ends with the rearrangement of their 12 descendants during formation of the somatic gonad primordium in the L2 stage (Kimble and Hirsh 1979). After the somatic primordium has formed, the 2 distal tip cells (DTCs) remain positioned at each distal end to lead extension of the gonad arms and nurture the germline stem cells, while the remaining somatic cells have coalesced in the proximal region. These cells include 3 ventral uterine precursor cells (VUs), which remain quiescent until the L3 stage, and a single terminally differentiated anchor cell (AC), a specialized ventral uterine cell that organizes subsequent development of the uterus and vulva. While the somatic primordium is forming, 4 proximal cells—2 α and 2 β cells (Fig. 1a)—initially have the potential to be the AC (Seydoux *et al.* 1990). The β cells soon lose the potential to be an AC and invariably become VUs, while the 2 α cells remain bipotential until LIN-12/Notch-mediated lateral specification resolves which will be the AC (Kimble 1981; Seydoux and Greenwald 1989; Seydoux *et al.* 1990).

Loss of *lin-12* activity has different consequences for α and β cell fate specification. In *lin-12(0)* mutants, both α cells become ACs, indicating that the αVU fate depends on *lin-12* activity. However, the βVU fate does not strictly depend on *lin-12* activity; instead, the requirement is heat-sensitive, such that in a *lin-12* null [*lin-12(0)*] mutant, many β cells become ACs at higher temperatures while at lower temperatures, most β cells become VUs (Sallee *et al.* 2015). Furthermore, in *lin-12(0)* mutants, β cells retain the potential to be ACs much longer than in a wild-type hermaphrodite, well into the L3 stage (Seydoux *et al.* 1990; Sallee *et al.* 2015). The difference in AC potential between α and β cells also appears influenced by POP-1/TCF, which accumulates differentially in α and β cells (Sallee *et al.* 2015). 

The sole *C. elegans* E protein, HLH-2, plays 2 distinct roles during ventral uterine fate patterning: *hlh-2* is first required to endow the 2 α and 2 β cells with the potential to be the AC and then to drive expression of the gene encoding the Notch ligand, *lag-2*, during the AC/VU decision by the α cells (Karp and Greenwald 2003, 2004). E proteins are obligate dimers, and for all of these roles, HLH-2 appears to function as a homodimer (Sallee and Greenwald 2015). In addition, the pattern of HLH-2 protein accumulation in the proximal region of the developing gonad reflects these different roles: initially expressed in the 2 α and 2 β cells, transgenic or endogenously tagged HLH-2 becomes restricted first to the 2 α cells, and then finally to the AC (Karp and Greenwald, 2003; Sallee and Greenwald, 2015; Attner *et al.* 2019; schematized in Fig. 1a). Moreover, the temporal expression of HLH-2 in the parents of the α cells also influences their fates: the first parent that expresses HLH-2 appears to endow its α daughter with an edge in LIN-12 activation (Attner *et al.* 2019). The regulation of HLH-2 expression and stability therefore appears intimately linked with a robust and reproducible cell fate patterning outcome.

Two inputs into regulation of HLH-2 expression and stability during ventral uterine fate patterning have been described thus far. First, a regulatory element called “*hlh-2*prox” is necessary and sufficient to drive expression in the α and β cells and their parents; deletion of this element results in all 4 cells becoming VUs because they are never endowed with the potential to be an AC (Sallee and Greenwald 2015; Attner *et al.* 2019). Second, HLH-2 is post-translationally degraded in VUs (Sallee and Greenwald 2015). Moreover, dimerization drives turnover in the VUs because mutations in the dimerization domain lead to stabilization of HLH-2 in the VUs; remarkably, the human E2A proteins E12 and E47 display similar dimerization-driven degradation in VUs, suggesting that this regulatory mechanism is conserved. This dimerization-driven degradation process may depend on proteasome activity, since depletion of proteasome components stabilizes HLH-2 in VUs (Sallee and Greenwald 2015). Proteasome-dependent degradation of HLH-2 was hypothesized to be part of a negative feedback loop that helps resolve the AC/VU decision, in which activation of LIN-12 leads to degradation of HLH-2, the transcription factor that drives *lag-2* expression in the AC (Karp and Greenwald 2003; Sallee and Greenwald 2015).

Here, we describe further investigations into the regulation of HLH-2 expression, stability, and activity during ventral uterine fate patterning. Our analysis indicates that *lin-12* activity leads to degradation of HLH-2 in the α cells, providing evidence for the hypothesized connection in the negative feedback loop in the presumptive αVU. We also show that *lin-12* activity leads to degradation of HLH-2 in the β cells, suggesting that ectopic stabilization of HLH-2 in β cells in a *lin-12(0)* background may cause β cells to adopt the AC fate. We performed a targeted RNA interference (RNAi) screen of 232 ubiquitin-related genes and identified 7 genes whose depletion ectopically stabilized HLH-2 in VUs; however, stabilization of HLH-2 in a *lin-12(+)* background caused by depletion of these ubiquitin-related genes did not alter the number of ACs, suggesting that *lin-12* activity normally opposes *hlh-2* activity in VUs by an additional mechanism apart from simply causing HLH-2 degradation. We also provide evidence for *lin-12*-independent downregulation of *hlh-2* transcription in β cells, suggesting that the putative POP-1/TCF input may contribute to the difference in potential between α and β cells at this level.

## Materials and methods

### *Caenorhabditis elegans* alleles and transgenes

Strains were grown and maintained under standard conditions, and experiments were performed at different temperatures as detailed below. The full genotypes of all strains used in this study are listed in Supplementary Table 1. All strains used in this study contain the single-copy insertion transgene *arTi145[ckb-3p::mCherry-h2b]* to mark somatic gonad cells with the red fluorescent protein mCherry (Attner *et al.* 2019).

Two engineered alleles of *hlh-2* were described in Attner *et al.* (2019): *hlh-2{ar623[gfp::hlh-2(+)]},* a knock-in allele that encodes GFP::HLH-2(+), and *hlh-2(ar614)*, hereafter called *hlh-2(*Δ*prox)*, a proximal gonad-specific null allele due to a deletion of a regulatory element in the *hlh-2* upstream regulatory region.

Strains containing the *lin-12(0)* molecular null allele *lin-12(n941)* (Greenwald *et al.* 1983; Wen and Greenwald 1999) were maintained using *arEx576*, an extrachromosomal rescuing array expressing *lin-12(+)* and other fluorescent coinjection markers, such that *lin-12(0)* homozygotes could be obtained by loss of the array (Sallee *et al.* 2015).

*nre-1(hd20) lin-15b(hd126)* was used to increase sensitivity to RNAi (Schmitz *et al.* 2007).

*arTi1* [*hlh-2prox::gfp*] is a single-copy transcriptional reporter generated by cloning the *hlh-2prox* element (Sallee and Greenwald 2015) upstream of *gfp*, followed by an *unc-54* 3’ UTR. The transgene was inserted into the genome on chromosome X using the transposon-based miniMos system (Frøkjær-Jensen *et al.* 2014).

*arIs51[cdh-3p::gfp]* (Karp and Greenwald 2003) and *qyIs176 [zmp-1(50-51)p::mCherry::moeABD]* (Schindler and Sherwood 2011) were used as markers for AC fate. *arIs51* and a similar *zmp-1*-based transgene, have been found to have the same fidelity as markers for AC fate (X. Karp and I.G., unpublished observations, cited in Karp and Greenwald 2004).

Transgenes generated for this study to test a potential kinase-regulated site are described below.

### Microscopy

Unless otherwise specified, we mounted larvae from plates onto 4% agarose pads and immobilized them using 10 mM levamisole. We used the *ckb-3p::mCherry-h2b* marker to confirm that each scored animal was at the appropriate developmental stage. For assessing AC marker expression and for the primary RNAi screen, animals were scored by eye using a 63x Plan-Apo objective lens on a Zeiss Axio Imager D1 microscope, using an X-Cite 120Q light source for illumination. For all other experiments, a spinning disc confocal equipped with a dual camera system was used to capture images. When both fluorescent proteins were imaged, GFP and mCherry fluorescence were captured simultaneously. A 488-nm, 100-mW laser was used to excite GFP and a 561- nm, 75-mW laser was used to excite mCherry. Exposure times for specific experiments are noted below. Imaging parameters associated with the RNAi screen are described along with other methods pertaining to the screen.

#### Quantifying endogenous GFP::HLH-2 levels and scoring AC number in lin-12(+) and lin-12(0) backgrounds

Eggs from the strains GS9790 and GS9814 were prepared using a standard bleaching protocol (Stiernagle 2006) and pipetted in the absence of food into an Erlenmeyer flask containing 10 ml of M9 buffer to induce L1 arrest. After shaking for 24–36 h at 20°C, L1 larvae were placed onto NGM plates seeded with OP50 bacteria. Plates were then transferred to 20°C and left to grow: 25 h for GS9790, 22 h for GS9814.

When scoring GS9790 animals, we ensured that coinjection markers associated with the extrachromosomal array *arEx576* were lost so that we only scored *lin-12(0)* larvae. We imaged endogenous GFP::HLH-2 using confocal microscopy as described above, at a 300-ms exposure time for GFP::HLH-2 and a 500-ms exposure time for mCherry-H2B.

#### Scoring AC number

We let the same strains grow until the larvae had reached at least the mid-L3 stage (34 h for GS9790, 31 h for GS9814) which we defined as the “Pn.px” stage of vulval development. Animals from the Pn.px stage to early invagination were scored for AC number. By this stage, a cell that expressed the AC basement membrane invasion marker *qyIs176 [zmp-1(50-51)p::mCherry::moeABD]* and retained stable GFP::HLH-2 expression was considered an AC.

#### Quantification of GFP::HLH-2 expression in GS9790 and GS9814

Image analyses were performed using Fiji (Schindelin *et al.* 2012). Sum z-projections of each α and β cell nucleus were generated based on *ckb-3p::mCherry-h2b* expression. For a given α or β nucleus, its *ckb-3p::mCherry-h2b* expression was used to draw a segmentation boundary; the expression level of GFP::HLH-2 was acquired by measuring GFP integrated intensity inside the boundary. To correct for background, GFP integrated intensity was measured in a region with the same dimensions of the original segmentation boundary, but outside the larva. This value was subtracted from the nucleus’ original integrated intensity value to give the corrected value.

#### Imaging hlh-2prox transcription in α and β cells

GS8981 and GS8982 eggs were synchronized at L1 arrest as described above. After shaking for 24–36 h at 20°C, L1 larvae were placed onto plates seeded with OP50 and grown for 18 to 21 h at 25°C. Confocal imaging of larvae was performed as described above. We imaged *hlh-2prox::gfp* at 250-ms exposure and *ckb-3p::mCherry-h2b* at 500-ms exposure. Z-stacks spanning the 4 α and β cells and/or their descendants were collected with slices taken at 260-nm intervals.

### RNAi screen of ubiquitin-related genes and validation of candidates

#### Ubiquitin-related gene library

The 232 conserved ubiquitin-related genes screened in this study were identified as follows. A list of *C. elegans* ubiquitin-related genes identified by Du *et al.* (2015) was analyzed using OrthoList 2 (Kim *et al.* 2018), resulting in a list of 239 conserved ubiquitin-related genes. We added the E2 enzyme *use-1* (Wang and Zhao 2019) and 7 members of the ring-between-ring (RBR) E3 ligase family (Eisenhaber *et al.* 2007) based on the literature (*ari-1.1, 1.2, 1.3, 1.4; ari-2; pdr-1; Y49F6B.9*), for a total of 247 conserved ubiquitin-related genes. We then analyzed the 260 clones covering 232 of the conserved ubiquitin-related genes that were available in the Ahringer library (including its supplement) (Kamath *et al.* 2003) and the Vidal library (Rual *et al.* 2004), covering ∼94% of conserved ubiquitin-related genes in *C. elegans*.

#### RNAi treatment

For each round of screening; *lacZ* RNAi served as a negative control to assess stabilization of GFP::HLH-2 by each of 10 experimental RNAi clones; *lin-12* RNAi served as a positive control for the RNAi conditions. Eggs from GS8995 animals were synchronized at L1 arrest as described above. We pipetted larvae onto RNAi feeding plates (Ahringer 2006) seeded with a feeding RNAi strain. Plates were placed at 25°C for 26 h before scoring.

#### Identifying candidate genes

Our primary screen was for the presence of GFP::HLH-2 in more than 1 cell. We mounted larvae onto 2% agarose pads and immobilized them with 10 mM levamisole. For each clone, we scored the number of α or β cells expressing visible GFP::HLH-2 in ≥20 individuals at this stage. Under these imaging and growth conditions, we found that GFP::HLH-2 was always bright in the presumptive AC but in some experiments was dimly visible in >1 cell even in the *lacZ(RNAi)* negative control. We attribute this to the fact that the endogenous GFP::HLH-2 is much brighter than transgenes used in previous work and the timing was not quite right to have reached the stage at which complete downregulation would have occurred in most animals. To be conservative, we only scored experiments for which ≥50% had only one cell in which GFP::HLH-2 was present in the negative *lacZ(RNAi)* control. In addition, we only scored experiments for which *lin-12* RNAi produced >50% of animals with supernumerary cells expressing GFP::HLH-2, indicating that the RNAi plates successfully induced expression of dsRNA.

We used “strict” and “lenient” criteria to identify potential regulators of GFP::HLH-2 stability. A gene met the “strict” criterion if the average number of somatic gonad cells with stable GFP::HLH-2 per worm was ≥2, suggesting that RNAi treatment stabilized GFP::HLH-2 at a high penetrance. A gene met the lenient criterion if (1) at least one animal scored had stable GFP::HLH-2 in all 4 α and β cells and (2) at least half of the population scored had >1 cell containing stable GFP::HLH-2, potentially allowing for more subtle stabilization to be recognized. After the initial screen, RNAi treatments fitting either of the 2 criteria were repeated in triplicate. Any candidate that did not meet either criterion upon repetition was removed from the candidate gene list.

#### Quantifying GFP::HLH-2 after candidate gene RNAi

RNAi was performed by an identical protocol as during the initial screen. However, the identity of each RNAi treatment was blinded except for the positive control, *lin-12* RNAi, which we used to ensure that the RNAi plates were properly inducing dsRNA expression. Larvae were imaged using confocal microscopy as described above. We imaged endogenous GFP::HLH-2 at 250-ms exposure and mCherry-H2B at 250-ms exposure. Z-stacks consisting of 50 slices were taken, with slices taken at 260-nm intervals. For each genotype, a “Blank” image was also taken using the same settings for the purposes of flatfielding.

Z-stacks were processed using a pipeline described in de la Cova *et al.* (2017). Using *ckb-3p::mCherry-h2b* as a segmentation marker, each α and β cell nucleus was identified and the 5 slices of each cell containing the highest integrated intensity of mCherry fluorescence. The integrated intensity values from the GFP channel of those slices were summed to produce the final intensity level of GFP::HLH-2 in each nucleus. There is no correction to remove background fluorescence in this pipeline, which makes it relatively insensitive to detect dim signals and may account for the candidates that appeared to be positive by qualitative criteria but not when quantified in this manner.

To facilitate comparisons between cells treated with different RNAi, we named the α and β cells as follows: the α cell with the highest GFP::HLH-2 fluorescence in a given animal was named “α_1_,” the other α cell α_2_, and the β cells β_1_ (sister of α_1_), and β_2_ (sister of α_2_). In some cases, individual cells or entire animals were excluded from quantification due to poor segmentation of α or β cell nuclei. This usually occurred for one of 3 reasons: (1) both α cells were so physically close together in a slice that they were segmented as an individual cell, (2) expression of *ckb-3p::mCherry-h2b* was not uniform in a given nucleus, so a single nucleus was segmented into multiple objects, or (3) the *ckb-3p::mCherry-h2b* signal was not strong enough compared to background to properly segment.

#### Assessing AC/VU phenotypes caused by RNAi of candidate genes

The strain GS9740 [*arTi145*; *arIs51*; *nre-1(hd120) lin-15b(hd126)*] was used to assess whether knockdown of candidate genes altered the AC/VU decision. In the proximal somatic gonad in the late L2/early L3 stage, *arIs51[cdh-3p::gfp]* is normally expressed only in the AC; we therefore scored for zero or >1 GFP-expressing cell. The RNAi protocol above was used except that treated larvae were grown for 32 h instead of 26, as *cdh-3p::gfp* is not visible until after the AC/VU decision is completed in late L2. For each clone, we scored the number of ACs by *cdh-3p::gfp* expression in ≥20 animals. The *lacZ* RNAi and *lin-12* RNAi were again used controls as described above.

### Testing a potential kinase-regulated site for effects on GFP::HLH-2 activity and stability

We created transgenes that encoded wild-type or mutant GFP::HLH-2 proteins by cloning into pWZ111, a vector designed for CRISPR/Cas9-mediated single-copy insertion at the ttTi4348 locus (Pani and Goldstein 2018). A ∼5.2-kb 5’ *hlh-2* regulatory sequence was cloned from the plasmid pMS49 (Sallee and Greenwald 2015), *gfp* and first exon of *hlh-2* were cloned from the plasmid pMS175 (Sallee and Greenwald 2015), and the remaining *hlh-2* sequences, including introns and 3’ UTR, were amplified from lysed *C. elegans* N2 gDNA. These fragments were assembled with pWZ111 using a standard Gibson assembly protocol (Gibson 2011). Desired SNPs were synthesized using primer overhangs. The constructs were inserted into the ttTi4348 locus using CRISPR/Cas9 (Pani and Goldstein 2018). *arSi113* and *arSi93* are independent but identical transgenes expressing HLH-2(+) [*hlh-2p::gfp::hlh-2(gDNA)::hlh-2 3’ UTR*]. *arSi101* contains the mutation *hlh-2(T341A)*, creating a putative phospho-null residue at the T341 position. *arSi103* and *arSi104* contain the mutation *hlh-2(T341D)*, a phospho-mimetic mutation at the T341 position. We evaluated the rescuing ability and stability of GFP::HLH-2 proteins produced from these transgenes in a WT and *hlh-2(*Δ*prox)* background.

We examined the ability of mutant HLH-2 proteins to rescue the completely penetrant Vulvaless (Vul) and Egg laying-defective (Egl) phenotype caused by the proximal gonad-specific *hlh-2(0)* allele *hlh-2(ar614)* [*hlh-2(*Δ*prox)*] (Attner *et al.* 2019). We placed a single individual of each genotype onto a plate, then let them self-fertilize and propagate. We then scored whether the progeny of each individual was Egl (based on the aggregation of eggs within an animal due to its inability to lay eggs) or underwent normal vulval and uterine development. Phenotypes were scored using a benchtop dissection scope.

To assess protein stability, eggs were collected using a standard bleaching protocol (Stiernagle 2006), placed onto NGM plates seeded with OP50, and grown at 25°C for 21 h. Larvae were imaged using confocal microscopy as described above, Both GFP and mCherry were imaged at 600-ms exposure. Z-stacks were collected with slices taken at 260-nm intervals for quantitation. In each animal, we scored (1) the number of α or β cells with stable GFP::HLH-2 and (2) whether there was 1 cell with significantly higher qualitative expression of GFP::HLH-2.

## Results

### *lin-12* activity promotes degradation of HLH-2 in α and β cells

In wild-type hermaphrodites, the α cells interact via LIN-12 to select which becomes the AC and which becomes the αVU, while the β cells invariably become VUs. As HLH-2 promotes expression of *lag-2*, the gene encoding the ligand for LIN-12 in the AC/VU decision, and many other genes important for AC function that are not expressed in VUs (Karp and Greenwald 2003; Hwang and Sternberg 2004), *lin-12* activity was proposed to negatively regulate degradation of HLH-2 in the αVU as part of a negative feedback mechanism to prevent ectopic AC fate (Karp and Greenwald 2003; Sallee and Greenwald 2015). HLH-2 is also degraded in the βVUs (Sallee and Greenwald 2015). We tested whether *lin-12* promotes HLH-2 degradation by quantifying the level of endogenous GFP::HLH-2 in the α and β cells in *lin-12(+)* and *lin-12(n941)*, a likely molecular null allele [hereafter, *lin-12(0)*].

We constructed 2 strains, both containing *hlh-2(ar623[gfp::hlh-2])* and the AC marker *qyIs176[zmp-1p::mCherry::moeABD]*, but differing in their *lin-12* genotype, one *lin-12(+)* and the other *lin-12(0)*. We quantified the endogenous GFP::HLH-2 expression levels in the α and β cells in the somatic gonad primordium in the L2 stage in both strains using photomicrographs captured on a confocal microscope (e.g. Fig. 1b) and determined the proportion of ectopic ACs generated (Fig. 1c). The proportion of ectopic ACs agrees with previously published work (Sallee *et al.* 2015). The difference in GFP::HLH-2 levels between the inferred ACs and αVUs in the *lin-12(+)* background is also shown (Fig. 1d).

We made 3 salient observations regarding the role of *lin-12* in GFP::HLH-2 stability. (1) GFP::HLH-2 expression was higher in α cells, which are fated to be ACs, in a *lin-12(0)* background compared to α cells in a *lin-12(+)* background. Even when excluding α cells with the lower level of GFP::HLH-2 in a given animal, likely VUs, from this analysis, there is still a significant difference between ACs of the 2 genotypes (Fig. 1e). This observation is consistent with *lin-12* activation promoting HLH-2 degradation while the α cells interact with each other to resolve which will become the AC, in accordance with the hypothesized negative feedback loop. (2) GFP::HLH-2 expression in βVUs was significantly higher in *lin-12(0)* than in *lin-12(+)* hermaphrodites (Fig. 1f). We note that we corrected for β cells that become ACs in the *lin-12(0)* background based on the proportion of β cells that adopted the AC fate (Fig. 1c) and excluded the 10% of β cells expressing the highest level of GFP::HLH-2 expression from this assessment (Fig. 1f). Thus, we conclude that *lin-12* activity contributes to HLH-2 degradation in βVUs, even though *lin-12* activity is not required for them to adopt the VU fate. (3) In both genotypes, the level of GFP::HLH-2 in β cells is lower than in α cells. Of particular note, the αVU has a significantly higher level of GFP::HLH-2 fluorescence than the βVUs, so the difference is not attributable to cell fate (Fig. 1g). This observation suggests a *lin-12-*independent level of regulation that distinguishes α from β cells.

### Negative regulation of *hlh-2* transcription in β cells

**Fig. 2.**
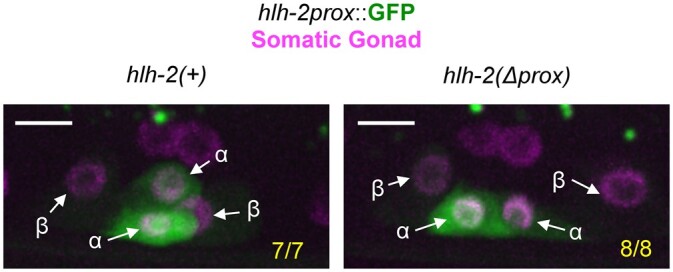
Transcription of *hlh-2prox* is negatively regulated in β cells independent of *lin-12* and *hlh-2* activity. Left, the transcriptional reporter *arTi1[hlh-2prox::GFP]* is initially expressed in the α and β cells (not shown) and becomes visibly restricted to the α cells. Right, *arTi1* expression is still restricted to the α cells in the background of *hlh-2(*Δ*prox)*, which has a deletion of a regulatory element that abrogates HLH-2 expression specifically in the α and β cells and their parents. This mutation leads to loss of LIN-12 expression in α and β cells, and all 4 α and β cells become VUs. Thus, restriction of transcriptional reporter to the α cells is not dependent on *hlh-2* or *lin-12* activity. *n* = 7 for *hlh-2(+)*, *n* = 8 for *hlh-2(*Δ*prox)*. All images are maximum projections of z-stacks collected using a spinning disc confocal microscope. Scale bars are 5 μm.

The *hlh-2* “prox” regulatory element is both necessary and sufficient for *hlh-2* expression in α and β cells (Sallee and Greenwald 2015; Attner *et al.* 2019). Deletion of this element from the endogenous gene results in a proximal gonad-specific null allele *hlh-2(*Δ*prox)*. In this background, the α and β cells never acquire the potential to be an AC and always become VUs, and do not express *lin-12* and *lag-2*. We constructed a transcriptional reporter derived from this element, *arTi1 [hlh-2prox::GFP]* (see *Materials and Methods*), and observed visible GFP expression in the α and β cells and their parents in an otherwise wild-type background, with expression becoming dimmer first in βVUs and then in the αVU (Sallee and Greenwald 2015). When we examined the expression of this reporter in the *hlh-2(*Δ*prox)* background, we observed that GFP was brighter in α cells, where it is readily apparent, than in β cells, where it is essentially undetectable (Fig. 2), even though the α and β cells no longer differ in their developmental potential. This observation suggests that *hlh-2* transcription is negatively regulated in β cells, and may account for the *lin-12*-independent level of regulation that distinguishes α from β cells inferred above (Fig. 1g) and for the POP-1-influenced early loss of AC potential by the β cells (Sallee *et al.* 2015).

### Identification of negative regulators of HLH-2 stability

Depletion of *hlh-2* activity in the L1 stage leads to loss of AC potential and a 0 AC phenotype, whereas depletion during the AC/VU decision leads to a failure of the AC/VU decision and a 2 AC phenotype (Karp and Greenwald 2004). HLH-2 is degraded in presumptive VUs, so to determine if it is sufficient to drive all 4 cells to be ACs, and/or to drive both α cells to be VUs, we need to protect it from degradation in a *lin-12(+)* background. Although HLH-2 may be stabilized in presumptive VUs by mutations that compromise its ability to dimerize (Sallee and Greenwald 2015), such mutations also abrogate its activity as a transcription factor, and therefore preclude analyzing the consequences of stabilizing HLH-2 on cell fate.

**Fig. 3.**
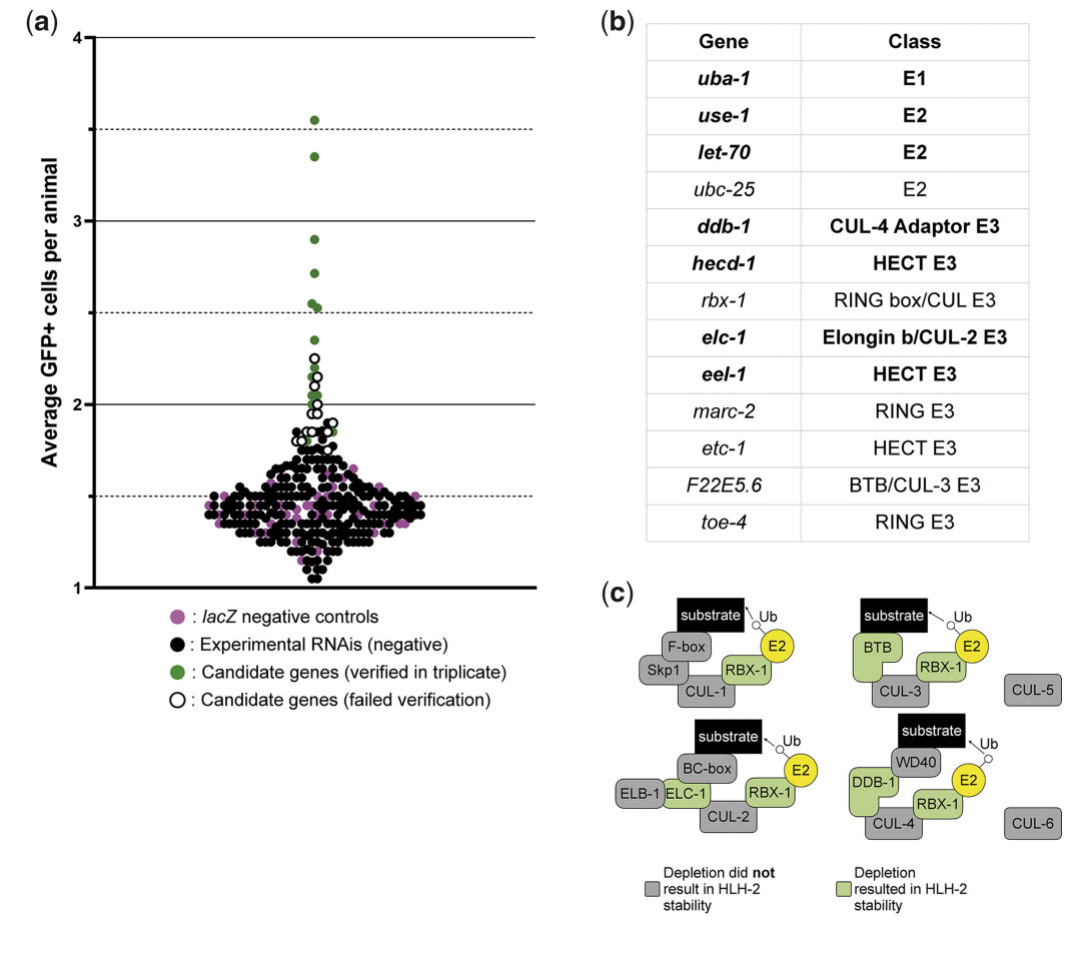
An RNAi screen of conserved ubiquitin-related genes identified potential regulators of HLH-2 stability in α and β cells. a) We screened 232 ubiquitin-related genes conserved in humans (see *Materials and Methods*) for stabilization of endogenous GFP::HLH-2 after RNAi. This set of genes included the sole E1 enzyme (*uba-1*), 25 E2 enzymes, 204 E3 ligases, the Nedd8 conjugating enzyme *ubc-12*, and the SUMO-conjugating enzyme *ubc-9*. Each circle represents ≥20 larvae scored after treatment by one of the 260 clones scored during the screen. Magenta circles represent the negative control *lacZ*, black circles represent clones that did not meet either criterion for a hit (see *Materials and Methods* for details), white circles outlined in black represent clones that initially met one or both criteria for a hit but did not recapitulate those results upon replication, and green circles represent the 14 clones encoding 13 candidate genes that met one or both criteria for a hit and recapitulated those results upon repetition. Each round of screening included a *lacZ* negative control, a *lin-12(RNAi)* positive control to ensure that the RNAi plates and conditions were functional (not shown), and 10 experimental genes. Each circle is positioned on the *y*-axis based on the average number of GFP+ cells in the ≥20 larvae scored. See *Materials and Methods* for further details. b) The thirteen genes that were considered *bona fide* candidates after rescreening in triplicate (see Supplementary Fig. 1). The genes in bold were deemed as the “stronger” candidates after fluorescence was quantified (see Fig. 4). c) Many of the candidate genes identified are known to be subunits of Cullin-based complexes. Cartoons representing the canonical Cullin-based complexes are depicted here, with candidate genes shown to reproducibly stabilize GFP::HLH-2 upon RNAi depletion colored in green. There are 6 *cullin* genes in *C. elegans*, but individual *cullin* RNAi depletions did not result in GFP::HLH-2 stability.

As an alternative approach, we performed an RNAi screen for trans-acting factors that would stabilize endogenous GFP::HLH-2(+) in a *lin-12(+)* background (see *Materials and Methods*). Because we knew that RNAi-mediated depletion of proteasome components results in stabilization of GFP::HLH-2 in VUs, and that human E2A proteins expressed in *C. elegans* are regulated in a similar manner (Sallee and Greenwald 2015), we screened 232 ubiquitin-related genes conserved in humans (see *Materials and Methods*). We identified thirteen genes for which RNAi caused visible ectopic stabilization of GFP::HLH-2 (Fig. 3): the sole E1 enzyme in *C. elegans*, *uba-1*; 3 E2 enzymes (*let-70*, *ubc-25*, and *use-1*); and 9 E3 ligases (*ddb-1*, *eel-1*, *elc-1*, *etc-1*, *F22E5.6*, *hecd-1*, *marc-2*, *rbx-1*, and *toe-4*). Four of the genes identified are components of Cullin-based E3 ligase complexes (Fig. 3c), but RNAi-depletion of individual *cullin* genes did not stabilize GFP::HLH-2.

**Fig. 4.**
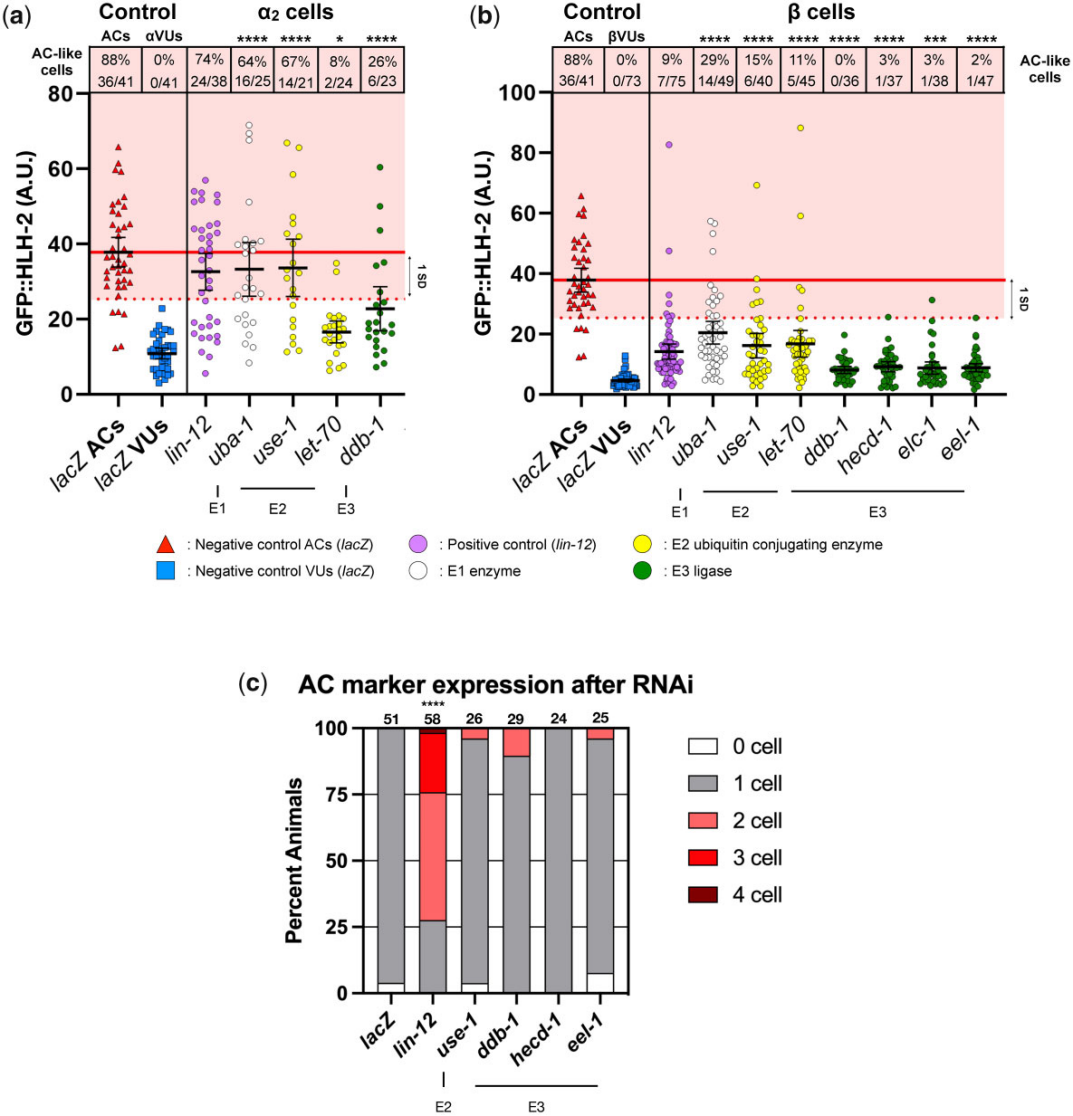
Depletion of candidate genes does not result in supernumerary ACs despite AC-like GFP::HLH-2 levels. GFP::HLH-2 fluorescence was quantified as described in *Methods and Materials*. As most individuals have a single AC after RNAi treatment, we assigned the cell with the highest level of GFP::HLH-2 in each larva, α_1,_ as the presumptive AC and assumed the other α cell, α_2_, is the αVU. Statistical comparisons were made to the *lacZ* negative control. a) GFP::HLH-2 level in α_2_. 4/13 RNAi treatments resulted in a significant increase in GFP::HLH-2 levels in α_2_: *uba-1*, *use-1*, *let-70*, and *ddb-1*. b) GFP::HLH-2 level in β cells. 7/13 RNAi treatments resulted in significant increases in GFP::HLH-2 levels in β cells—the 4 aforementioned genes, plus *elc-1*, *eel-1*, and *hecd-1*. In panels a and b), we identified α and β cells, respectively, that had “AC-like” GFP::HLH-2 levels. We found the mean GFP::HLH-2 fluorescence (horizontal red line) of ACs scored in the negative control (red triangles). We characterized any cell with GFP::HLH-2 fluoresence greater than 1 SD below the mean as cells with AC-like levels of GFP::HLH-2 (pink background). The percent of cells with this level of GFP::HLH-2 (excluding the presumptive AC, α_1_) is shown in the table above the graph. Statistical tests for both analyses are Kruskal–Wallis tests with Dunn’s multiple comparison tests. *P < 0.05, **P < 0.01, ***P < 0.001, ****P < 0.0001. Black lines represent the mean GFP::HLH-2 fluorescence for each RNAi treatment, and error bars are 95% confidence intervals. Candidate genes that did not result in significant differences in GFP::HLH-2 levels based on our statistical analyses were excluded from this figure (see Supplementary Fig. 2). c) To find whether ectopic HLH-2 stability resulted in AC/VU defects, we treated larvae expressing an AC marker with candidate gene RNAi. To ensure that any 0 AC defects were due to a failure to specify an AC and not due to a delay in gonad development, we only scored animals that had progressed past the 12-cell stage of somatic gonad development. Only *lin-12* RNAi, the positive control, resulted in a statistically significant number of AC/VU defects (1 AC vs. non-1 AC, Fisher’s exact test, ****P < 0.0001). Note that 3 RNAi treatments, *elc-1*, *let-70*, and *uba-1* resulted in arrest of the somatic gonad at the 12-cell stage, so we were unable to score AC marker expression using these staging conditions. Number of animals scored after each RNAi treatment is listed above each bar.

We then quantified levels of endogenous GFP::HLH-2 stabilization by confocal imaging. We compared corresponding cells to assess if depletion of a candidate gene stabilized GFP::HLH-2 preferentially in the α cells, β cells, or both. We therefore named the α cell with the highest GFP::HLH-2 fluorescence in a given animal “α_1_,” and also assumed that it was an AC (see also below); we named the other α cell α_2_, and the β cells β_1_ (sister of α_1_), and β_2_ (sister of α_2_). RNAi treatment of 4/13 candidate genes resulted in a statistically significant difference in GFP::HLH-2 levels in α_2_ compared to a *lacZ* RNAi control: the E1 *uba-1*; 2 E2s, *use-1* and *let-70*; and the E3 *ddb-1* (Fig. 4a). RNAi depletion of these 4 genes also resulted in a significant increase in GFP::HLH-2 levels in β cells, as did RNAi directed against 3 additional E3 enzymes: *hecd-1*, *elc-1*, and *eel-1* (Fig. 4b). We consider these 7 genes to be the strongest candidate genes identified by our screen and used them to assess the effects of ectopic stabilization of HLH-2(+) on cell fate in the next section.

### Ectopic stabilization of HLH-2(+) appears to be insufficient to alter the number of ACs in a *lin-12(+)* background

As described above, the loss-of-function phenotype of *hlh-2* affects the number of ACs, depending on the time of depletion: RNAi directed against *hlh-2* beginning in the L1 stage results in a 0 AC phenotype (both α cells become VUs) while RNAi directed against *hlh-2* in the L2 stage results in a 2 AC phenotype (both α cells become ACs) (Karp and Greenwald 2004). If stabilization of HLH-2 is sufficient to extend AC competence or promote AC fate, we might expect supernumerary ACs and consequently fewer VUs. However, stabilization of HLH-2 may also result in continued *lag-2* and *lin-12* expression in multiple cells, opposing AC fate and promoting VU fate, leading to a 0 AC phenotype. It is also possible that there could be a mixture of fates, depending on the dynamics or level of stabilization.

As in the *lin-12(0)* experiments above, we used a two-part approach. We first considered whether RNAi against any of the 7 stronger candidate genes stabilized endogenous GFP::HLH-2 in any α_2_ and/or β cells to the same level as an AC (the α_1_ cell). We defined the threshold for “AC-like” levels as 1 SD below the mean of GFP::HLH-2 fluorescence in ACs—any cell above this threshold was considered “AC-like.” Every presumptive VU scored in the negative control was below this threshold, while ∼88% of ACs were above, indicating that this is a conservative threshold. By this criterion, apart from the sole E1 component *uba-1*, the strongest effect seen was for the E2 *use-1*, which resulted in 67% (14/21) of α_2_ cells (Fig. 4a) and 15% (6/40) of β cells (Fig. 4b) falling above the threshold.

To assess whether ectopic stabilization of GFP::HLH-2 altered the number of ACs, we generated a strain that substituted the AC fate marker *arIs51 [cdh-3::gfp]* for GFP::HLH-2 but otherwise had the same genetic background (see *Materials and Methods*; Supplementary Table 1). To ensure that any defects we observed were not secondary consequences of developmental arrest—a particular concern for potential 0 AC defects that might result if arrest occurred before the AC/VU decision was completed—we only scored animals that had >12 somatic gonad cells, by which point an AC should have been specified. The E1 *uba-1*, the E2 *let-70*, and the E3 *elc-1* were excluded from this analysis because their development was arrested at the 12-cell stage by the RNAi treatment. None of the remaining 4 RNAi treatments resulted in significant AC/VU defects (Fig. 4c). Strikingly, *use-1* RNAi resulted in ectopic AC marker expression in only 1/26 animals, which by position appeared to be an α cell, whereas as described above, 67% (14/21) of α_2_ cells had a GFP::HLH-2 level above the AC threshold. Similarly, 0/52 β cells displayed ectopic AC marker expression, whereas 15% (6/40) had GFP::HLH-2 level above the AC threshold.

These observations suggest that ectopic HLH-2 stabilization itself is not sufficient to disrupt the resolution of the AC/VU decision by the α cells, and therefore that abrogation of the negative feedback loop resulting in HLH-2 degradation in the presumptive αVU is not sufficient to disrupt the AC/VU decision. They also suggest that ectopic HLH-2 stabilization alone is not sufficient to promote AC fate or otherwise alter the fates of the α or β cells in a *lin-12(+)* background.

### HLH-2 activity does not appear to be regulated by phosphorylation of T341

**Fig. 5.**
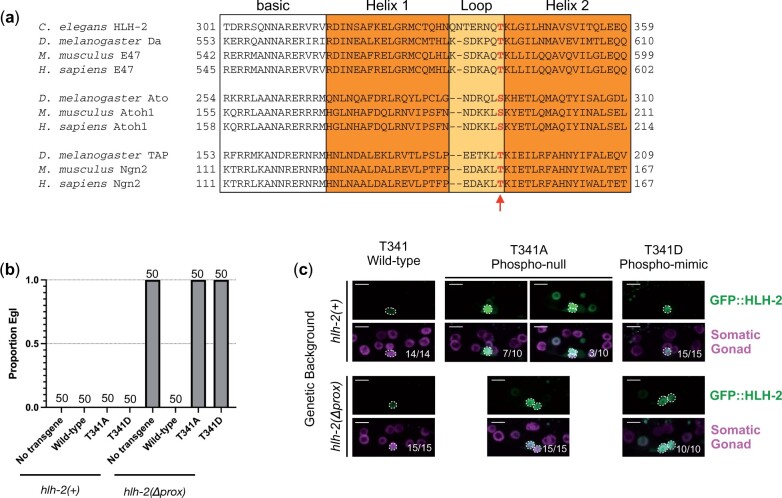
Analysis of mutations of the conserved residue T341. a) The bHLH domain of *C. elegans* HLH-2 aligned with the *Drosophila melanogaster*, *Mus musculus*, and *Homo sapiens* orthologs, as well as the bHLH domains of the class II bHLH proteins Ato and TAP, showing conservation of the T residue regulated by phosphorylation (Quan *et al.* 2016), shown in red and marked with a red arrow. b) Single-copy insertion transgenes at a defined site in the genome, expressing wild-type, phospho-mimetic (T341D), and phospho-null (T341A) GFP::HLH-2 mutant proteins, were examined in *hlh-2(+)* and *hlh-2(*Δ*prox)* backgrounds. The T341A and T341D mutations did not have a dominant phenotype in an *hlh-2(+)* background; furthermore, they were unable to rescue the egg-laying defect (Egl) of *hlh-2(*Δ*prox)*, indicating that these mutations compromise HLH-2 function. c) Representative maximum projections of z-stacks are shown with GFP::HLH-2 (green) alone above and GFP::HLH-2 and a somatic gonad marker (magenta) merged below. GFP::HLH-2(+) is stabilized and brightest in a single cell in both *hlh-2(+)* and *hlh-2(*Δ*prox)* backgrounds. The phospho-null (T341A) mutant protein is most often stabilized in a single cell in the *hlh-2(+)* background (7/10) but is sometimes bright in 2 cells (3/10). In an *hlh-2(*Δ*prox)* background, it is bright in 2 cells, the α cells. The phospho-mimetic (T341D) mutant protein is bright in a single cell in an *hlh-2(+)* background but is bright in 2 cells in an *hlh-2(*Δ*prox)* background. Cells with bright HLH-2 expression are circled by a white dotted line. Scale bars are 5 μm.

Certain Class II bHLH proteins can be inactivated by phosphorylation of a conserved serine/threonine residue at the junction of the loop and the second helix of the bHLH domain; mutation of this residue to A to abrogate phosphorylation results in constitutive activity, and mutation to D is phosphomimetic and prevents activation (Quan *et al.* 2016) (see Fig. 5a). Because ectopic HLH-2(+) stability after RNAi of ubiquitin-related genes did not result in an AC/VU defect in a *lin-12(+)* background, and T341 is in a corresponding position in HLH-2 (Fig. 5a), we tested whether phosphorylation of T341 deactivates HLH-2 homodimers.

We generated constructs that express GFP::HLH-2(+), a putative phopho-null mutant protein [GFP::HLH-2(T341A)] and a putative phospho-mimetic mutant protein [GFP::HLH-2(T341D)] and created single-copy insertion transgenes into a defined site on LGI (see *Materials and Methods*) so that they would be expressed at equivalent levels. If T341 regulates the activity of HLH-2 as it does Class II bHLH proteins, we would expect HLH-2[T341D], a putative phospho-mimetic mutation, to be inactive and HLH-2[T341A] to be active.

We tested the ability of these transgenes to rescue the fully penetrant 0 AC defect caused by the proximal gonad-specific null allele *hlh-2(*Δ*prox)*. The GFP::HLH-2(+) transgene fully rescued this defect, whereas neither mutant transgene had any rescuing activity (Fig. 5b), suggesting the mutant proteins are not active.

The pattern of protein accumulation suggests that mutation of T341 affected the ability of the proteins to dimerize: in the *hlh-2(*Δ*prox)* background, both GFP::HLH-2(T341D) and HLH-2(T341A) appeared equally bright in the 2 α cells and dimmer in β cells (Fig. 5c). All 4 of these cells are presumptive VUs, although they differ from wild-type VUs in never having had AC potential. We interpret these results as indicating that the mutant proteins compromise homodimerization and therefore prevent degradation in presumptive VUs. The T341 mutant HLH-2 monomers may be stable because HLH-2 homodimerization is required for its turnover (Sallee and Greenwald 2015), or because *lin-12*, which we hypothesize promotes HLH-2 degradation, is not expressed in a background in the absence of *hlh-2* activity (Attner *et al.* 2019). In either case, the lower levels of protein in β cells reflect lower transcription.

In addition, we tested the T341 mutants for dominant effects in an *hlh-2(+)* background, as might result if heterodimers with endogenous HLH-2(+) have dominant-negative or altered activity, but no dominant effects were observed (Fig. 5b). The pattern of protein accumulation of GFP::HLH-2(T341D) was identical to that of HLH-2(+) in this background (Fig. 5c). We did observe some animals (3/10) where GFP::HLH-2(T341A) accumulated in 2 cells. Because we did not observe any dominant effects in this strain, we again interpret this to mean that the T341A mutation somehow prohibits the mutant protein from dimerizing, thus resulting in its increased stability in some animals. Indeed, this stability pattern is reminiscent of the stability pattern of HLH domain mutants as described in Sallee *et al.* (2015), supporting this interpretation.

In sum, our analysis suggests that T341 does not regulate the activity of HLH-2 in the manner previously seen for Class II bHLH proteins, although it is possible that the specific amino acid substitutions that were tolerated by the Class II proteins to support the mechanism in heterodimers are not tolerated in HLH-2 homodimers.

## Discussion

The AC/VU decision is precise and robust: although 4 cells, 2 α cells and 2 β cells, are initially born with the potential to be the AC, wild-type hermaphrodites always have a single AC. The 2 β cells always become VUs, but unlike many cell fates in *C. elegans*, there is natural variability in which α cell becomes the AC: the 2 α cells interact with each other via LIN-12/Notch to ensure that only one becomes the AC and the other becomes a VU (reviewed in Greenwald 2012).

*hlh-2* is required for all aspects of AC development, endowing the α and β cells with the potential to be the AC, driving expression of the ligand for LIN-12 during the decision, and AC differentiation genes thereafter (Karp and Greenwald 2003, 2004; Hwang and Sternberg 2004; Hwang *et al.* 2007; Schindler and Sherwood 2011; Verghese *et al*. 2011; Bodofsky *et al.* 2018; Medwig-Kinney *et al.* 2020). We have provided evidence herein for a proposed negative feedback loop (Karp and Greenwald 2003; Sallee and Greenwald 2015) in which *lin-12* activity promotes HLH-2 degradation in VUs: when *lin-12(0)* and *lin-12(+)* hermaphrodites are compared, the average level of GFP::HLH-2 is higher in the 2 α cells, both of which are always ACs, and in the 2 β cells, which sometimes become ACs and sometimes become VUs, in *lin-12(0)* mutants.

However, by reducing the activity of trans-acting factors that act as negative regulators of HLH-2 stability, we also found that HLH-2 stabilization appears to be insufficient to promote the AC fate or otherwise alter the fates of the α or β cells in a *lin-12(+)* background. By contrast, multiple ACs are specified in a *lin-12(0)* background. Because individuals in the *lin-12(+)* background had a single AC, it is unlikely that *lin-12* activity was disrupted by depletion of these trans-acting factors.

We consider here 3 possible explanations for why stabilization of HLH-2 in presumptive VUs is not sufficient to transform them into ACs in the presence of LIN-12. The first possibility is that HLH-2 is inactivated as a consequence of LIN-12 activity in VUs. We explored a potential mechanism by which this might occur, based on the observation that certain Class II bHLH proteins can be inactivated by phosphorylation of a conserved serine/threonine residue at the junction of the loop and the second helix of the bHLH domain (Quan *et al.* 2016), but our analysis suggests that this mechanism does not account for our other observations. A second possible mechanism is that there is another factor (or factors) that works in conjunction with HLH-2 to confer AC potential and/or in the AC/VU decision, and this factor is also downregulated in response to LIN-12 activation but is not stabilized by the same RNAi treatments that stabilize HLH-2. If the missing component is a transcription factor, it is unlikely to be a Class II bHLH protein, as there is good evidence that HLH-2 does not have a Class II heterodimerization partner in the α and β cells (Sallee and Greenwald 2015; Sallee *et al.* 2017). A third possibility is that degradation of HLH-2 contributes to the robustness of the AC/VU decision but that even when HLH-2 is stabilized by RNAi targeting ubiquitin-related factors, there is still a sufficient difference between the α cells that can be amplified by feedback mechanisms implemented upon LIN-12 activation during the AC/VU decision.

We also identified 4 E3 enzymes as “strong” regulators of HLH-2 stability in α and β cells. We do not know whether there is a direct enzyme-substrate relationship with HLH-2 for any of the candidates that we identified, but it is provocative that all 4 have connections suggestive of roles in the AC/VU decision. *hecd-1* was previously shown to display genetic interactions consistent with a role as a positive regulator of *lin-12* activity in the AC/VU decision (Chen and Greenwald 2014). *eel-1* has been implicated in a negative feedback loop in the Wnt/β-catenin pathway (de Groot *et al.* 2014), of interest given the apparent role for POP-1/TCF in differential competence of the α and β cells (Sallee *et al.* 2015). Finally, *ddb-1* (Kim and Kipreos 2007) and *elc-1* (Merlet *et al.* 2010) are involved in cell cycle regulation, of interest given that the onset of HLH-2 expression in parent cells appears to be coupled to their cell cycle (Attner *et al.* 2019), and that HLH-2 is degraded in the VUs, cells that have not exited the cell cycle, but is stable in the AC, which is terminally differentiated. It will be interesting to explore these potential connections in future work, and to determine if there is coordination between these different ubiquitination pathways, as has been observed for the sequential actions of different complexes in regulating the level of CUL-1 in *C. elegans* (Dove *et al.* 2017).

## Data availability

The data underlying this article are available in the article and in its Supplementary material online.

## Supplementary Material

jkac028_Supplementary_Figure_S1

jkac028_Supplementary_Figure_S2

jkac028_Supplementary_Table_S1
