# Supplementary material for: Influences of HLH-2 stability on anchor cell fate specification during Caenorhabditis elegans gonadogenesis
Source: G3 (Bethesda). 2022 Feb 3;12(4):jkac028. doi: 10.1093/g3journal/jkac028 (PMC8982380; doi:10.1093/g3journal/jkac028)
Supplement: jkac028_Supplementary_Figure_S1 [file jkac028_supplementary_figure_s1.pdf]

A.

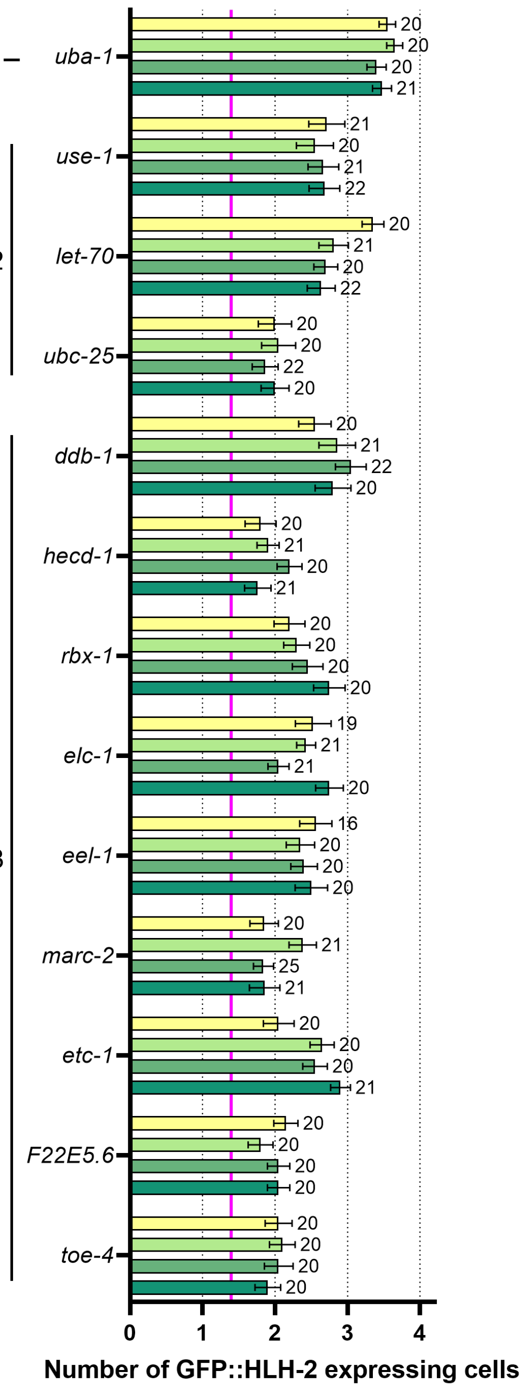

B.

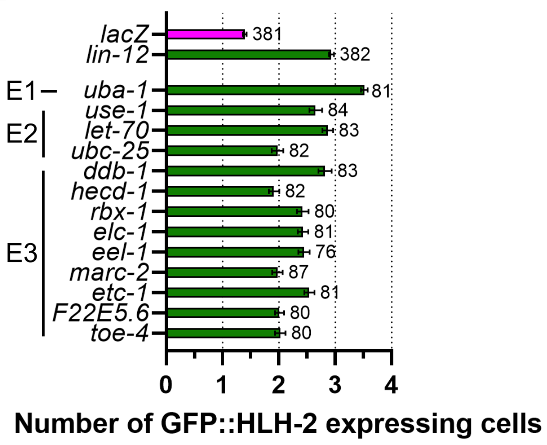

Figure S1. The thirteen genes that met criteria for candidate regulators of HLH-2 stability.

A) 13/26 candidates obtained after the initial round of screening emerged after rescreening in triplicate. Each bar represents one round of scoring, with the yellow bar representing the initial round scored during the screen, and green bars representing replicates. Error bars are 95% confidence intervals and the n for each round is shown next to each bar. The magenta line represents the mean of all negative control (lacZ) RNAi treatments for comparison.

B) Combined results shown in A. Every candidate gene RNAi treatment (green bars) resulted in a significantly increased number of GFP+ cells per animal compared to the negative control RNAi treatment, lacZ (magenta bar) (Kruskal-Wallis test with Dunn's multiple comparisons tests, $p < 0.0001$ for all RNAi treatments).
