# Supplementary material for: Influences of HLH-2 stability on anchor cell fate specification during Caenorhabditis elegans gonadogenesis
Source: G3 (Bethesda). 2022 Feb 3;12(4):jkac028. doi: 10.1093/g3journal/jkac028 (PMC8982380; doi:10.1093/g3journal/jkac028)
Supplement: jkac028_Supplementary_Figure_S2 [file jkac028_supplementary_figure_s2.pdf]

A.

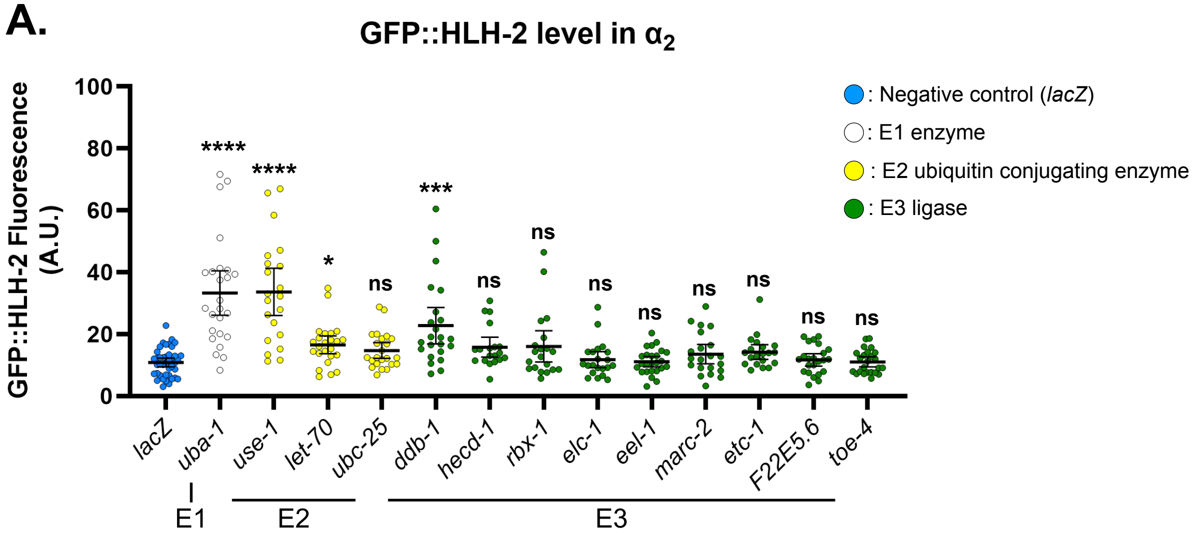


B.

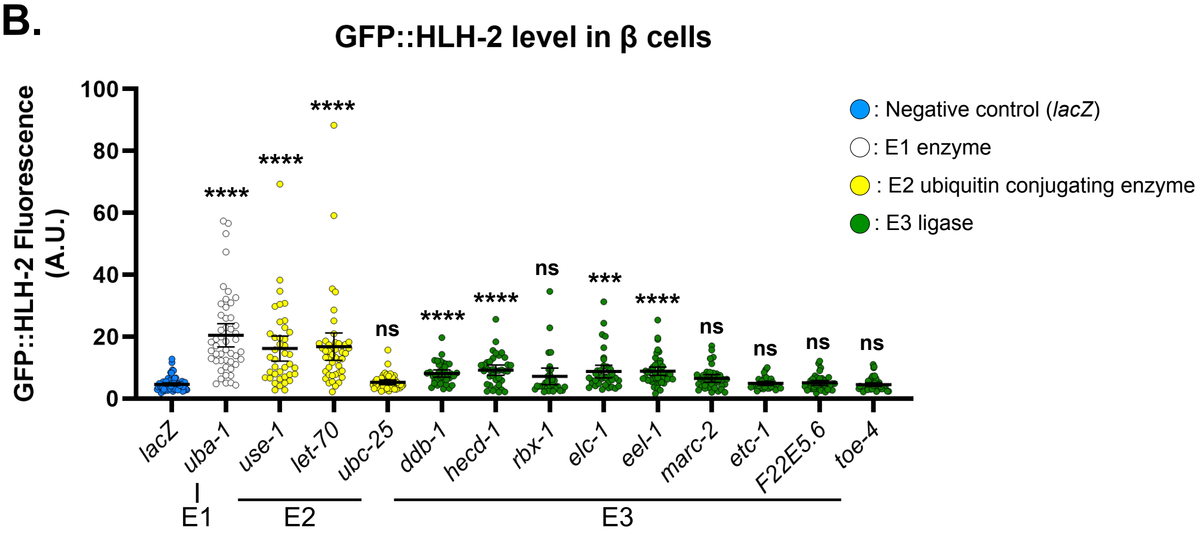


**Figure S2.** Quantitative analysis of GFP::HLH-2 levels after RNAi of candidate genes.

Data shown here are identical to that shown in Figure 4, but now includes candidate gene RNAi treatments that did not result in GFP::HLH-2 levels significantly different from the negative control based on our analysis.

A) GFP::HLH-2 level in $\alpha_2$ cells.

B) GFP::HLH-2 level in β cells. Statistical tests for both analyses are Kruskal-Wallis tests with Dunn's multiple comparison tests. * represents $p < 0.05$, ** represents $p < 0.01$, *** represents < 0.001, **** represents $p < 0.0001$. Black lines represent the mean GFP::HLH-2 fluorescence for each RNAi treatment, and error bars are 95% confidence intervals. We note that, although the remaining six genes identified by qualitative criteria (*etc-1*, *F22E5.6*, *marc-2*, *rbx-1*, *toe-4*, and *ubc-25*) did not result in quantitatively significant increases in GFP::HLH-2 levels by this analysis, we think it likely due to background fluorescence, which was not subtracted by the pipeline used for this analysis, suggesting that these RNAi treatments resulted in weaker stabilization.
