# Supplementary material for: Influences of HLH-2 stability on anchor cell fate specification during Caenorhabditis elegans gonadogenesis
Source: G3 (Bethesda). 2022 Feb 3;12(4):jkac028. doi: 10.1093/g3journal/jkac028 (PMC8982380; doi:10.1093/g3journal/jkac028)
Supplement: jkac028_Supplementary_Table_S1 [file jkac028_supplementary_table_s1.pdf]

Strain	Full Genotype	Interpreted Relevant Genotype*	Figure(s)
GS8513	*arTi145*	*ckb-3p::mCherry-h2b* (somatic gonad cell marker)	6B
GS8859	*hlh-2(ar614); arTi145*	α/β-specific *hlh-2(0)*	6B
GS8981	*arTi145*; *arTi1*	*hlh-2prox::gfp*	2
GS8982	*hlh-2(ar614); arTi145; arTi1*	α/β-specific *hlh-2(0)*; *hlh-2prox::gfp*	2
GS8995	*hlh-2(ar623)*; *arTi145*; *nre-1(hd120) lin-15b(hd126)*	*gfp::hlh-2*; RNAi sensitizer	3A-B, 4, 5A
GS9635	*arSi93 hlh-2(ar614); arTi145*	*hlh-2p::gfp::hlh-2(+); α/β-specific hlh-2(0)*	6B-C
GS9638	*arSi103; arTi145*	*hlh-2p::hlh-2(T341D)* (phosphomimetic)	6B-C
GS9639	*arSi101; arTi145*	*hlh-2p::hlh-2(T341A)* (phosphonull)	6B-C
GS9643	*arSi104 hlh-2(ar614); arTi145*	*hlh-2p::hlh-2(T341D), α/β-specific hlh-2(0)*	6B-C
GS9645	*arSi113; arTi145*	*hlh-2p::gfp::hlh-2(+)*	6B-C
GS9739	*arSi101 hlh-2(ar614); arTi145*	*hlh-2p::hlh-2(T341A), α/β-specific hlh-2(0)*	6B-C
GS9740	*arTi145; arIs51; nre-1(hd120) lin-15b(hd126)*	*ckb-3p::mCherry-h2b*; AC marker; RNAi sensitizer	5B
GS9790	*hlh-2(ar623); arTi145; lin-12(n941); qyIs176; arEx576*	GFP::HLH-2; *lin-12(0)*; AC marker; *Ex[lin-12(+)]*	1B-F
GS9814	*hlh-2(ar623); arTi145; qyIs176*	GFP::HLH-2; *AC marker*	1B-F

*all strains contain the somatic gonad marker
